# Supplementary material for: Complexome profiling of the Chlamydomonas psb28 mutant reveals TEF5 as an early PSII assembly factor
Source: Plant Cell. 2025 Mar 17;37(6):koaf055. doi: 10.1093/plcell/koaf055 (PMC12164590; doi:10.1093/plcell/koaf055)
Supplement: koaf055_Supplementary_Data [file koaf055_supplementary_data.zip › psb28_interactive.zip!/plots/intensity/qualityPlots/peptides/_Cre12.g540051.t1.1.html]

### Cre12.g540051.t1.1

Trivial name:   
SeqCoverage [median]: 11.70  
Mass: 35.30 kDa  
  
 

| Sequence | MissedCleavage | Mass [Da] | wtIntensity | muIntensity | Score |
| --- | --- | --- | --- | --- | --- |
| LERPPNDVVYLGPLSQQHK | 1 | 2189.16 | 0.00 | 1851.01 | 51.09 |
| TTTLLGNSR | 0 | 961.52 | 510.28 | 1056.59 | 87.48 |
| YTAASGQVEVR | 0 | 1179.59 | 2196.59 | 3954.54 | 106.40 |
|  |  |  | 2706.9 | 6862.1 |  |
